# 117 Assessing Burn Care Policies in Low- and Lower-Middle-Income Countries

**DOI:** 10.1093/jbcr/iraf019.117

**Published:** 2025-04-01

**Authors:** Alice Umutoni, Ulrick Kanmounye, Jonathan Ayeyi Nuamah, Elaf Abdelraheem, Ezekiel Adetoye, Theophilus Barasa, Sarah Okojie, Linda Efua Jackson, Emmanuel Baah Nyarko, Joshua Angaama, Piel Panther Kuol, Shirley Dadson, Jesse Kiprono Too, Heba Hassaballa Elsadig Abdelrahim, Ruben Ayala

**Affiliations:** Operation Smile International; University of Ghana Medical School; University of Ghana Medical School; Faculty of Medicine, University of Khartoum; Lagos State University Teaching Hospital; Kenyatta University; Lagos State University Teaching Hospital; University of Ghana Medical School; University of Ghana Medical School; University of Buea; Moi University; University of Ghana Medical School; Moi University; University of Medical Sciences and Technology; Operation Smile International

## Abstract

**Introduction:**

Burns account for an estimated 18 million disability-adjusted life years (DALYs) annually. Of note, the burden of burn-related health issues is not distributed equally, with low-income and lower-middle-income countries often lacking adequate health policies to address this issue. This study sought to identify burn care-related health policies and clinical guidelines in 26 low-income and 52 lower-middle-income countries.

**Methods:**

Ministry of Health (MoH) websites for 78 countries were searched for published burn care policies and clinical guidelines using English, French, Arabic, and Spanish keywords, languages the authors were fluent in. Seven countries were excluded as they lacked an MoH website. An additional eight countries were excluded as their websites employed languages in which the authors lacked proficiency, such as Portuguese, Nepali, Burmese, Lao, and Russian. Resources from 63 MoH websites were subsequently included. An eight-fold path framework for policy analysis was employed to evaluate the retrieved burn care policy.

**Results:**

100 documents, including policies, clinical guidelines, and research publications, were retrieved. Of these, 29 results were exclusive burn policy and clinical guidelines. These burn policies were implemented between 1976 and 2024, with clinical guidelines extending to 2027. 31% were clinical guidelines, and 69% were health care policies. Most policies focused on burn prevention and first aid, while clinical guidelines focused on burn emergencies.

**Conclusions:**

Despite the extensive burden of burning in these regions, burn care policies in low-income and lower-middle-income countries are still nonexistent in most countries. Based on these findings, we recommend intentional advocacy and support for comprehensive burn policy development and implementation in low-income and lower-middle-income countries.

**Applicability of Research to Practice:**

Low and middle-income countries bear a disproportionate share of the global burn injury burden. This research demonstrates a lack of comprehensive care policies and clinical burn management protocols. By drawing attention to these deficiencies, policymakers can be informed about the critical need to develop appropriate guidelines covering all aspects of burn care, from initial first aid to advanced treatment of severe cases. Additionally, this highlights the importance of addressing policies related to long-term follow-up care, emergency response, and the education of healthcare professionals in burn management.

**Funding for the Study:**

N/A

